# MetaPath Chat: multimodal generative artificial intelligence chatbot for clinical pathology

**DOI:** 10.1002/mco2.769

**Published:** 2024-10-10

**Authors:** Haizhu Chen, Ruichong Lin, Yu Yunfang

**Affiliations:** ^1^ Guangdong Provincial Key Laboratory of Malignant Tumor Epigenetics and Gene Regulation, Guangdong‐Hong Kong Joint Laboratory for RNA Medicine, Department of Medical Oncology Breast Tumor Centre, Sun Yat‐sen Memorial Hospital, Sun Yat‐sen University Guangzhou China; ^2^ Faculty of Innovation Engineering Macau University of Science and Technology Taipa Macao China; ^3^ Faculty of Medicine Macau University of Science and Technology Taipa Macao China

1

Recently, two pivotal studies, one published in *Nature* ^1^ and another in *Cell*,^2^ present groundbreaking advancements that are set to revolutionize artificial intelligence (AI) in pathology. The first study introduced PathChat, a multimodal generative AI assistant for human pathology.^1^ The second study unveiled TriPath, a weakly supervised AI model designed for analyzing three‐dimensional (3D) pathology samples and predicting patient outcomes.^2^ These findings highlight the potential of AI to revolutionize pathology by enhancing diagnostic and prognostic accuracy and enabling new forms of human–machine collaboration.

Lu et al.^1^ introduced PathChat, an AI assistant designed to aid pathologists in diagnostic workflows (Figure 1). PathChat integrated a vision‐language model that combined a pretrained vision encoder with a large language model, fine‐tuned on over 456,916 visual‐language instruction, encompassing 999,202 question–answer turns. The vision encoder, based on the UNI architecture, was pretrained on over 100 million histology image patches from over 100,000 slides and further refined with 1.18 million pathology image‐caption pairs.

**FIGURE 1:**
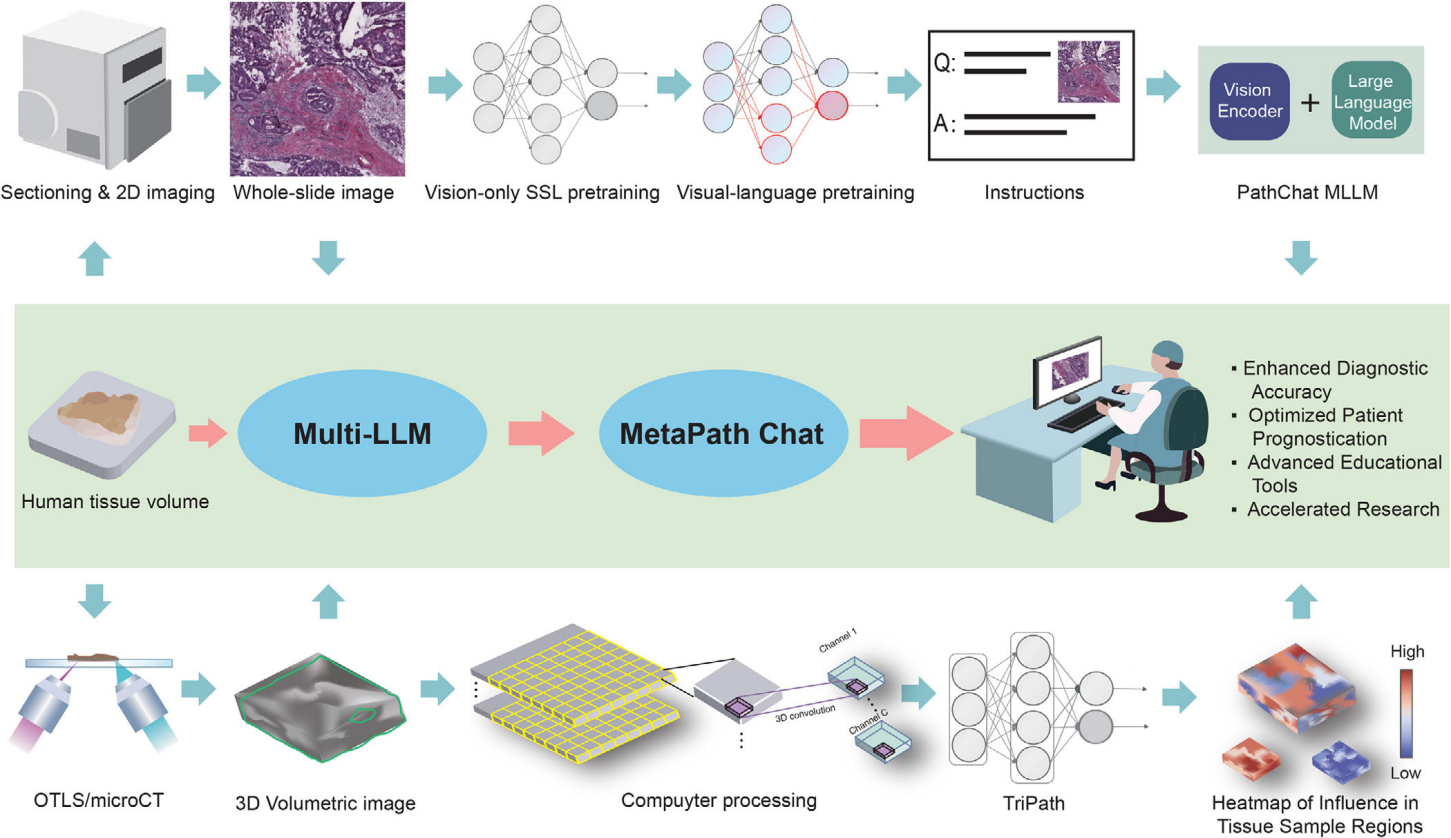
Workflow and application of transformative artificial intelligence (AI) models for enhanced diagnostic and prognostic accuracy in pathology. The top section illustrates the process of building a multimodal large language model (MLLM)‐based vision language AI assistant for pathology, named PathChat. Whole‐slide images generated from 2D imaging of human tissue volumes undergo vision‐only self‐supervised learning (SSL) pretraining, followed by visual‐language pretraining. This pretraining involves 456,916 instructions to develop a robust PathChat MLLM combining a vision encoder and a large language model, enabling question and answer functionalities. This integration enables the system to accurately respond to pathology‐related queries by analyzing both image and text inputs, thereby providing real‐time diagnostic support and reducing variability in clinical decision‐making. The bottom section shows the workflow for developing TriPath, a model specifically designed for analyzing 3D pathology structures. Human tissue volumes are imaged using Optical Projection Tomography (OTLS) or microcomputed tomography (microCT) to create volumetric images. These images are processed using 3D convolution techniques, which break down the data into smaller, manageable units that are analyzed to produce channel outputs. TriPath processes these data to create heatmaps, which visually represent regions within the tissue sample that have varying levels of diagnostic and prognostic significance. By leveraging the strengths of PathChat and TriPath, we propose the concept of a novel integrated model, MetaPath Chat. This AI expert system is designed to comprehensively process various forms of pathological images, with the potential to enhance diagnostic accuracy, optimize patient prognostication, provide advanced educational tools, and accelerate research in pathology.

The performance of PathChat was rigorously evaluated against state‐of‐the‐art multimodal AI assistants, including LLaVA 1.5, LLaVA‐Med, and GPT‐4 V.^1^ First, evaluations focused on multiple‐choice diagnostic questions using routine H&E whole slide images (WSIs) from both The Cancer Genome Atlas and an in‐house pathology archive, covering 54 diagnoses from 11 major pathology practices and organ sites. Evaluations were conducted in two settings: image‐only and image with clinical context. PathChat outperformed its counterparts in diagnostic accuracy for multiple‐choice questions. Specifically, in the image‐only setting, PathChat achieved a 78.1% accuracy (+52.4% vs. LLaVA 1.5 and +63.8% vs. LLaVA‐Med, both *p* < 0.001). In the image with clinical context setting, PathChat's accuracy improved to 89.5% (+39.0% vs. LLaVA 1.5 and +60.9% vs. LLaVA‐Med, both *p* < 0.001), demonstrating its ability to leverage multimodal information effectively. Moreover, PathChat outperformed GPT‐4 V in both image‐only (78.8 vs. 25%) and image with clinical context (90.5% vs. 63.5%) settings, highlighting its superior diagnostic accuracy.

Furthermore, Lu and colleagues^1^ assessed the ability of PathChat to generate coherent, clinically relevant responses to open‐ended pathology‐related questions. Seven expert pathologists ranked the responses of different models based on relevance, correctness, and explanation quality. PathChat produced more preferable responses compared with other multimodal large language models (MLLMs), with a median win rate of 56.5%, 67.7%, and 74.2%, respectively, against GPT‐4 V, LLaVA 1.5, and LLaVA‐Med. Importantly, PathChat also supported interactive, multiturn conversations, making it a versatile tool for education, research, and clinical decision‐making.

In discussing the clinical contributions of PathChat, Lu and colleagues^1^ also scrutinized its limitations and suggested directions for further research. The training data for PathChat, although extensive, were derived from retrospective datasets, which might contain outdated information. Consequently, continuous updates to the training data and model alignment with current practices are necessary to maintain accuracy and relevance. Moreover, future research could enhance PathChat's capabilities by extending support for WSIs, incorporating reinforcement learning from human feedback, and developing functionalities like precise counting or localization of objects within images.

In traditional histopathology, the reliance on 2D cross‐sections often fails to capture critical spatial information present in 3D structures. Song et al.^2^ addressed this limitation by developing TriPath, a deep learning model leveraging weakly supervised AI for analyzing 3D pathology samples (Figure 1). The weakly supervised AI has been confirmed to successfully identify critical pathological features with minimal manual labeling, showcasing performance on par with, and in some cases superior to, fully supervised methods. TriPath employed a combination of convolutional neural networks and transformer architectures to process volumetric data, segmenting large tissues into smaller 2D or 3D patches and summarizing them into low‐dimensional feature vectors for patient‐level risk prediction. Trained on a large dataset of annotated 3D pathology samples, TriPath demonstrated high accuracy in identifying various pathological conditions.

The study tested the utility of TriPath for risk stratification using prostate cancer specimens imaged with difference 3D modalities, including open‐top light‐sheet microscopy and microcomputed tomography.^2^ TriPath consistently outperformed traditional 2D slice‐based approaches and even clinical baselines assessed by certified pathologists, effectively reducing variability in risk prediction caused by heterogeneous tissue structures.

The implementation of TriPath could streamline diagnostic processes, enabling more efficient and accurate analysis of 3D samples. Additionally, by minimizing the need for large volumes of labeled data, this approach could significantly reduce the resources required for AI training, making it accessible to more institutions. However, TriPath relied on high‐quality serial sections, which might not always be available. Additionally, the substantial computational resources required for 3D reconstruction may limit its widespread adoption in resource‐constrained settings. Future research should focus on improving the accessibility and efficiency of 3D reconstruction techniques, developing methods to handle lower‐quality samples and reducing computational demands.

The application of novel AI technologies into clinical practice holds immense potential.^3^, ^4^ However, pathology practices currently face several challenges, including diagnostic variability, time‐intensive manual slide examinations, and the limitations of analyzing 2D cross‐sections. These challenges not only impact diagnostic accuracy but can also delay the treatment decisions. The integration of novel AI technologies like PathChat and TriPath into clinical practice offers significant potential to address these challenges. As a multimodal AI assistant, PathChat enhances diagnostic accuracy by integrating visual and textual data. Meanwhile, TriPath, as a weakly supervised AI model, focused on analyzing 3D pathology samples, providing more comprehensive tissue analysis, facilitating risk stratification, and reducing diagnostic variability.

The implementation of PathChat and TriPath in clinical pathology can optimize workflow by automating routine diagnostic tasks, enabling pathologists to concentrate on more challenging cases. This technological synergy not only increases efficiency but also addresses variability in diagnoses caused by limited experience among pathologists. Additionally, these novel AI technologies contribute to reducing diagnostic turnaround times, facilitating quicker treatment decisions, and ultimately improving patient outcomes. Future research should focus on further refining these models, incorporating more diverse datasets, and exploring real‐world applications. Additionally, adding additional data types, such as proteomics, genomics, and radiology, could create even more comprehensive and accurate diagnostic tools.^5^

Moreover, integrating multimodal generative AI with weakly supervised AI may create a synergistic effect, leading to more accurate and comprehensive tools. Herein, we propose the concept of a novel model, MetaPath Chat, based on MLLMs (Figure 1). Such an integrated system is designed to comprehensively process various forms of pathological images and can leverage the strengths of each technology: the ability of multimodal generative AI to provide context‐aware and interactive responses, and the efficiency of weakly supervised AI in extracting meaningful features from large datasets without extensive annotations. It may offer the potential for more accurate diagnostics, optimized patient prognostication, enhanced educational tools, and accelerated research. Future research should strive to achieve the construction and application of MetaPath Chat, leveraging the strengths of integrated models to enhance performance and broaden its applicability.

Altogether, these two studies represent significant advancements in the application of AI to pathology. PathChat provides a powerful multimodal tool that enhances diagnostic workflows and supports educational and research activities, while TriPath brings a new level of detail and accuracy to 3D tissue analysis. Future research should focus on making these technologies more accessible and efficient, ensuring their broad implementation to improve patient outcomes and advance our understanding of various diseases.

## AUTHOR CONTRIBUTIONS

Haizhu Chen and Ruichong Lin conceived, drafted, and revised the manuscript. Yunfang Yu conceived and revised the manuscript. All authors have read and approved the final manuscript.

## CONFLICT OF INTEREST STATEMENT

The authors declare no conflict of interest.

## ETHICS STATEMENT

Not available.

## Data Availability

Not available.
